# Potential Therapeutic Effects of *Citrus hystrix* DC and Its Bioactive Compounds on Metabolic Disorders

**DOI:** 10.3390/ph15020167

**Published:** 2022-01-29

**Authors:** Hawa Nordin Siti, Suhaila Mohamed, Yusof Kamisah

**Affiliations:** 1 Department of Basic Medical Sciences, Faculty of Medicine, Universiti Sultan Zainal Abidin, Kuala Terengganu 20400, Malaysia; hawanordin@gmail.com; 2 UPM-MAKNA Cancer Research Laboratory, Institute of Bioscience, Universiti Putra Malaysia, Serdang 43400, Malaysia; mohamed.suhaila@gmail.com; 3 Department of Pharmacology, Faculty of Medicine, Universiti Kebangsaan Malaysia, Kuala Lumpur 56000, Malaysia

**Keywords:** kaffir lime, diabetes mellitus, hypertension, dyslipidemia, obesity

## Abstract

Metabolic disorders like diabetes mellitus, hypertension, dyslipidemia, and obesity are major medical problems globally. The incidence of these disorders has increased tremendously in recent years. Studies have demonstrated that plants with antioxidant and anti-inflammatory properties have beneficial effects on these disorders. One of these plants is *Citrus hystrix* DC, commonly known as kaffir lime. This review aims to present updates on the progress of research regarding the use of *C. hystrix* in metabolic disorders. Phytochemical compounds, including β-pinene, sabinene, citronellal, and citronellol, have been detected in the plant; and its extract exhibited potential antidiabetic, antihyperlipidemic and anti-obesity activity, as well as prevention of development of hypertension. These beneficial properties may be attributable to the presence of bioactive compounds which have therapeutic potential in treating these metabolic disorders. The compounds have the potential to be developed as candidate drugs. This review will assist in validating the regulatory role of the extract and its bioactive compounds on metabolic disorders, thus expediting future research in the area.

## 1. Introduction

Metabolic disorder, which includes hypertension, diabetes mellitus, dyslipidemia, and obesity, represents a major global health concern due to increased morbidity and mortality. It occurs due to disturbance in normal metabolic process leading to redox and energy imbalance [1]. The incidence of metabolic disorders increased during the COVID-19 lockdown due to lack of activities and physical exercise, as well as increased intake of homemade food rich in fat [2]. Many factors contribute to the development of this disorder, including an unhealthy diet, sedentary lifestyle, lack of physical exercise, and smoking [1]. Several therapeutic goals have been established to educate high-risk individuals to modify their lifestyle to slow down or prevent the progression of metabolic disorders.

Oxidative stress and inflammation play a major role in the development and progression of metabolic disorders [1,3]. Therefore, it has been theorized that plant extracts with antioxidant and anti-inflammatory properties could have beneficial effects on patients with metabolic disorders. Animal studies have shown that plant extracts such as those of *Parkia speciosa* Hassk., which is rich in flavonoids, confer protective effects against hypertension [4], while *Ganoderma lucidum* proteoglycans protect against diabetes [5], and açaí seed extract rich in proanthocyanidins protects against dyslipidemia [6]. *Citrus hystrix* DC extract has shown protective effects against diabetes [7], hypertension [8], and dyslipidemia [9].

**Figure 1:**
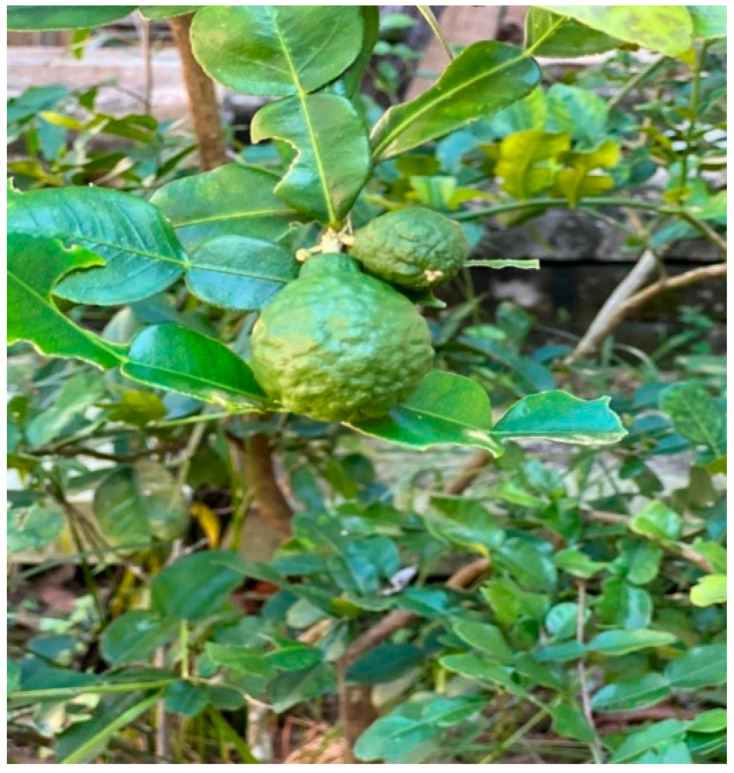
The plant of *Citrus hystrix* DC.

*Citrus hystrix* (Figure 1), also known as kaffir lime or makrut lime, also goes by the following names: *Citrus auraria* Michel, *Citrus echinata* SaintLager, *Citrus hyalopulpa* Tanaka, and *Citrus kerrii* (Swingle) Tanaka [10]. It is a flowering, shrubby plant in the family Rutaceae that grows 3 to 6 m high and is indigenous to tropical Southeast Asia, southern China, and northeastern India [10,11]. It bears green, warty, and bumpy fruits. The leaves and fruits are often used as spices in Asian cooking [12]. Many bioactive compounds from the plant have been studied for their therapeutic potential in improving the symptoms of metabolic disorders in animal studies. Therefore, the current review aims to gather up-to-date information on the pharmacological properties of *Citrus hystrix* and its bioactive compounds, and its effects on metabolic syndrome. The aim is for the findings to promote further research into the plant’s bioactive compounds to better understand their effects on medical disorders.

## 2. Literature Search

**Figure 2:**
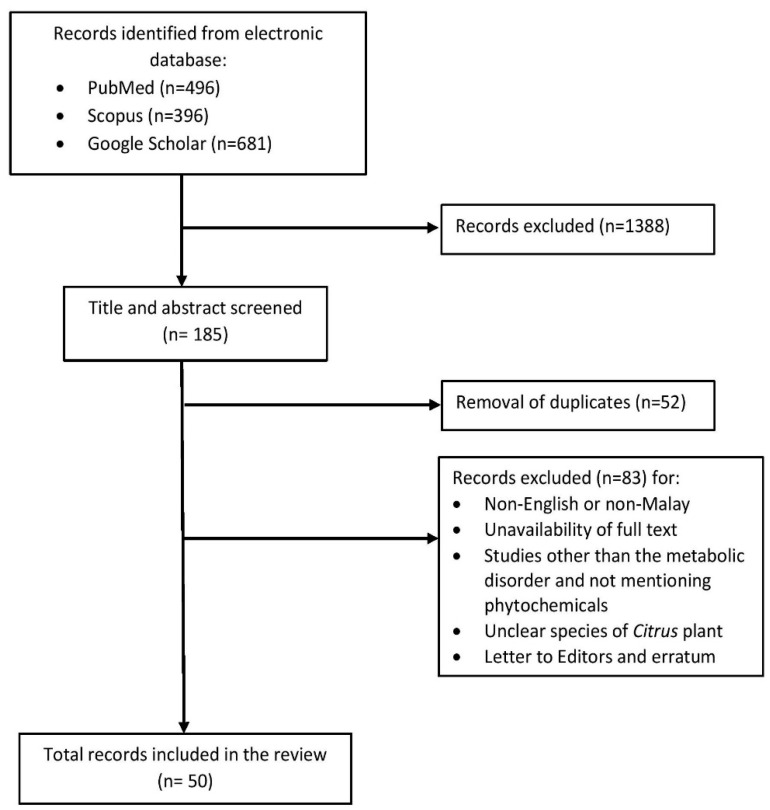
The flow of literature search.

A literature search was conducted in three electronic databases which were PubMed, Scopus and Google Scholar by using the following sets of search terms: “*Citrus hystrix*” OR “kaffir lime” AND “metabolic disorder” OR “metabolic syndrome” OR “hyperlipidemia” OR “hypertension” OR “diabetes” OR “obesity” OR “pharmacokinetics” OR “toxicity” OR “ethnobotanical” OR “medicinal use” OR “bioactive compound” OR “phytochemical”. Original articles and reviews published in English or Malay were included in this review. Articles published in other languages, not available full-text, studies with unclear *Citrus* species, and studies other than the metabolic disorder were excluded. Based on the inclusion and exclusion criteria, fifty articles were finally included in the review (Figure 2).

## 3. Traditional Medicinal Uses

The fruits, leaves, and rind of *C. hystrix* are the most common parts traditionally used to reduce the severity of certain illnesses (Table 1). The fruits are used for the treatment of stomachache by hilly Tripura tribes in northeastern India [11], while the leaves and fruits are both used in steam-bathing for postpartum mothers, to relieve headache, rheumatism, fever, and to treat diabetes mellitus in North Sumatra, Indonesia [13]. In Malaysia, the fruits are used in hair shampoo to decrease dandruff and to promote hair growth [10]. The leaves and fruits are also used to boost sexual performance [13] and to treat hypertension, heart disease, and diarrhea [14,15].

## 4. Phytochemical Properties

Various phytochemical compounds have been detected in the leaves, roots, fruits, and rind of *C. hystrix* (Table 2). Terpenoids are the major compounds identified in the leaves of the plant, while coumarins are predominantly found in the roots. The leaves also contain phytosterol and flavonoids. The rind extract, which is rich in flavonoids, possesses high antioxidant. It also demonstrates lipase-inhibiting activity which is beneficial for the treatment of obesity, angiotensin-converting enzyme-inhibiting property for the management of hypertension, moderate inhibiting activity against α-amylase and α-glucosidase which could be useful in diabetes, as well as inhibitions against acetylcholinesterase, butyrylcholinesterase, and β-secretase-1 which are favorable in the treatment of Alzheimer’s disease [19].

**Figure 3:**
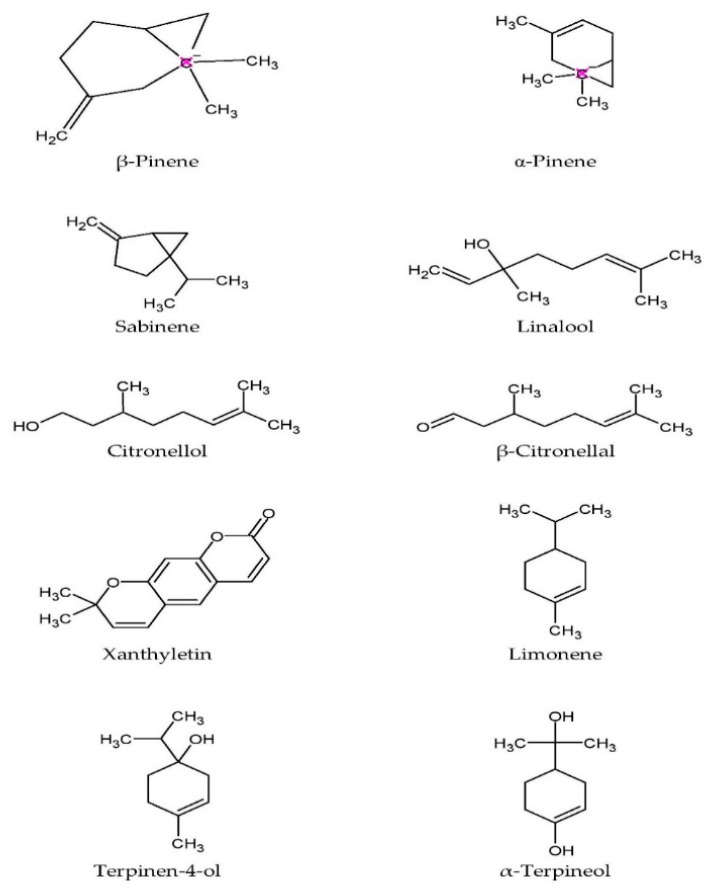
Molecular structure of major phytochemical compounds in *C. hystrix*.

Essential oils extracted from the twigs and leaves contain monoterpenes like citronellal, citronellol, linalool, sabinene, and limonene [35,36,37,38], while the major compounds in the oil from the rind are β-pinene, sabinene, limonene, citronellal, α-pinene, and terpinen-4-ol [38]. The oil from the leaves was noted to be inactive against *Staphylococcus aureus*, *Staphylococcus epidermidis*, *Bacillus subtilis*, *Escherichia coli*, *and Klebsiella pneumoniae*, but was moderately effective in inhibiting the fungal growth of *Candida albicans*, *Saccharomyces cerevisiae*, and *Cryptococcus neoformans* [36]. The antifungal activity of the leaf oil could be attributable to the presence of oxygenated monoterpenes [40]. Figure 3 shows the chemical structure of major phytochemical compounds found in the plant.

## 5. Effects on Diabetes

Diabetes mellitus is one of the metabolic disorders that has become a global public health burden. Type 1 diabetes occurs due to pancreatic β-cell damage leading to impaired insulin release, while type 2 diabetes occurs due to insulin resistance and is commonly associated with obesity [41]. Oxidative stress and inflammation are involved in the pathogenesis of the disorder [42]. Based on this knowledge, various plant extracts with antioxidant and anti-inflammatory properties, including *C. hystrix*, are being studied to assess potential protective effects against diabetes, in particular, type 2 diabetes.

Abirami et al. [43] (Table 3) demonstrated in an in vitro study that the powdered rind and pulp of *C. hystrix* exhibited higher concentration-dependent glucose adsorption capacity than that of xanthan and guar gum, and that the capacity was augmented with increasing concentration of glucose. This finding suggests that the extract could decrease postprandial glucose levels. The extract also decreased glucose dialysis retardation index and starch digestibility [43], suggesting a delay in glucose absorption possibly due to lower starch assimilation in the gastrointestinal tract. The extract could be beneficial in decreasing postprandial glycemic and insulinemic response in type 2 diabetics, thus controlling the uptake of glucose. However, the active phytochemical compounds responsible for the activity were not identified. The functional groups responsible for the glucose absorbency were most likely hydroxyl group and possibly methyl ester group of galacturonic acid detected in the *C. hystrix* extract [43].

Another in vitro study investigated the potential antidiabetic effects of different fractions of *C. hystrix* rind extract in ethanol [44]. In the study, the ethyl acetate fraction and water residue demonstrated a significant α-amylase-inhibiting activity comparable to metformin, a biguanide antihyperglycemic drug. The ethyl acetate fraction exhibited better α-glucosidase-inhibiting activity than the water residue (Table 3). The inhibition exhibited by the ethyl acetate fraction was similar to that of acarbose, an α-glucosidase inhibitor. However, the hexane fraction did not exhibit inhibitory activity against either enzyme. It appears that the increased polarity of certain compounds conferred the beneficial properties. α-Amylase hydrolyzes starch into disaccharides [48], while α-glucosidase converts disaccharides into monosaccharides before absorption into a portal vein [49]. In terms of antioxidant properties, the ethyl acetate fraction exhibited the highest activity followed by the water residue and hexane fraction. Both the ethyl acetate fraction and water residue contained alkaloids, saponins, tannins, phenolics, and flavonoids, while the hexane fraction contained alkaloids, phenolics, and flavonoids. It is possible that either certain phytochemical compounds in the ethyl acetate fraction and water residue exhibited the positive effects on glucose metabolism or that the compounds had interacted synergistically. Further studies should be performed to elucidate the responsible compounds. Fruit juice from the plant also demonstrated similar inhibitory effects on both enzymes (α-amylase and α-glucosidase) [45]. Ethanol rind extract exhibited a high inhibitory effect on α-amylase activity as compared with the low activity seen in the aqueous and ethyl acetate extracts [46]. This finding suggests that phytochemical compounds which have more polarity than ethyl acetate but less than the aqueous solution were responsible for the effect.

To the best of our knowledge, only a study had investigated in vivo effects of *C. hystrix* extract. Drinking 150 or 300 mg/kg body weight of *C. hystrix* leaf extract in solution with water lowered fasting blood glucose in streptozotocin-induced diabetic rats [7]. The beneficial effects of the extract led to a significant reduction in the incidence of cataracts in the rats, a phenomenon which was believed to be associated with reductions in oxidative stress and inflammation, indicated by lower levels of malondialdehyde, prostaglandin E2, and tumor necrosis factor-α (TNF-α); attenuated vascular permeability was also observed due to decreased levels of vascular endothelial growth factor. In the study, bioactive compounds were identified in the extract, namely apiin, apigetrin, saponarin, rutin, diosmin, hesperidin, and xanthotoxol [7]. These compounds have been shown to possess anti-inflammatory and antioxidant activity [50,51,52,53]. Further studies should be conducted to confirm the potential antidiabetic effects of the compounds in vivo as well as their mechanisms of action. The effects of the compounds on poly (ADP-ribose) polymerase and aldose reductase activity in the lens should be investigated as these enzymes are elevated in diabetic cataracts [54,55].

In a study by Rekasih et al. [47], a functional food drink was created containing various medicinal plants: *Orthosiphon aristatus* (Blume) Miq., *Zingiber officinale* Roscoe, *Caesalpinia sappan* L., *Curcuma xanthorriza* Roxb., *Citrus limon* (L.) Osbeck, *Citrus aurantifolia* (Christm.) Swingle, and *C. hystrix*. The drink contained 1% *C. hystrix* fruit juice and was administered at a dose of 18.2 mL/kg body weight to streptozotocin-induced diabetic rats for two weeks in ready-to-drink, microencapsulated, or nanoencapsulated formulations. The exact concentration of the *C. hystrix* juice in the drink could not be determined as the yield percentage was not reported. All formulations significantly reduced fasting blood glucose and elevated pancreatic β-cell and Langerhans islet viability in the rats, but the improvements were more significant in the microencapsulated and nanoencapsulated formulations. Encapsulation was believed to have increased the contact surface and improved the bioavailability of the bioactive phytocompounds in the drink [56]. The study demonstrated possible synergistic interactions among the components of the drink. Possible mechanisms of the hypoglycemic effects were not elucidated.

**Figure 4:**
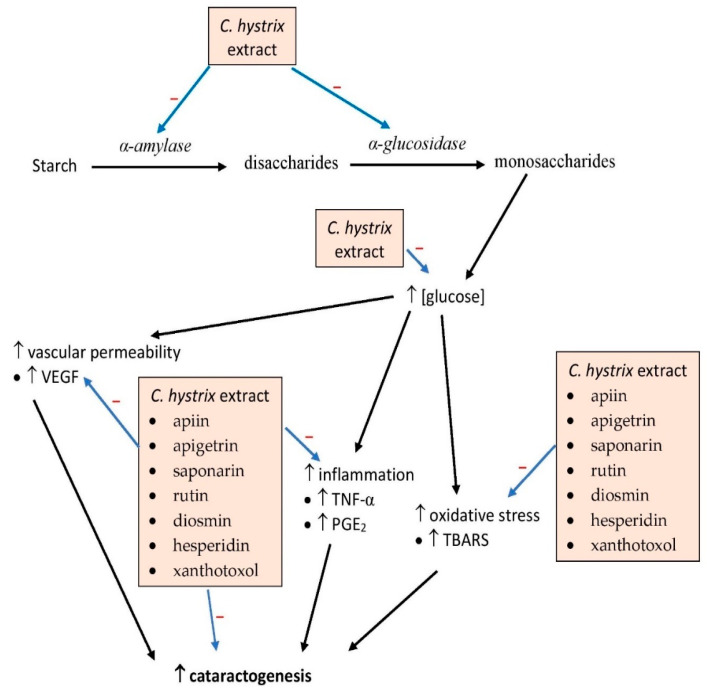
Possible sites of action of *C. hystrix* and its bioactive compounds in diabetes. PGE2, prostaglandin E2; TBARS, thiobarbituric acid reactive substance; TNF-α, tumor necrosis factor-α; VEGF, vascular endothelial growth factor; →, induces, → (with −), inhibits.

Collectively, almost all parts of *C. hystrix* possess antidiabetic properties most likely attributable to the plant’s flavonoid content. These compounds may act by suppressing α-amylase and α-glucosidase activities, in addition to possessing antioxidant and anti-inflammatory properties (Figure 4). The effect of the compounds on the mitogen-activated protein kinase pathway involved in the cellular inflammatory response, apoptotic and phosphatidylinositol 3-kinase/protein kinase B/mammalian target of rapamycin (PI3K/Akt/mTOR) pathways, gene and protein expression of glucose transporters and insulin receptors should be further elucidated. The PI3K/Akt/mTOR pathway is involved in intracellular cell cycle signaling, especially apoptosis and proliferation, and may also be involved in the viability of pancreatic β-cell and Langerhans islets. However, no clinical study has been conducted so far to assess the effects of the extract on diabetes. The phytochemical compounds have the potential for development as drugs for the treatment of type 2 diabetes mellitus.

## 6. Effects on Hypertension

Hypertension is the most common cardiovascular disease. Several mechanisms have been proposed for its pathogenesis, including impairment of renal salt and water handling and increased formation of angiotensin II involving the renin–angiotensin–aldosterone system (RAAS), abnormalities in the sympathetic system, and elevation of vascular oxidative stress and inflammation [57,58].

The aqueous extract of *C. hystrix* leaves demonstrated good angiotensin-converting enzyme (ACE) inhibition activity (>90%) in vitro [59]. The enzyme metabolizes angiotensin I into angiotensin II which then promotes aldosterone release, leading to salt and water retention [4]. There was a strong correlation between ACE inhibition and total phenolic content in the extract [59]. Plants rich in polyphenols were reported to exhibit ACE inhibitory effects [4,60]. The inhibitory effect exhibited by the *C. hystrix* extract suggests that its bioactive compounds could be potential candidates for treating hypertension. However, the compounds were not identified in the study and the effect of the extract was not compared with a positive control to validate its activity [59].

Heated oil has been reported to raise blood pressure in rats [61,62]. Heating causes chemical changes like thermal oxidation and polymerization in the oil, leading to a configuration change in the fatty acids from *cis* to *trans* [61]. Addition of *C. hystrix* leaf extract at 1% into frying oils that were heated five and ten times reduced peroxide levels and increased the total phenolic content of the oils [63,64] (Table 4). Reduction in the oxidized content of the oil by the addition of the extract prevented the elevation of blood pressure in rats that were fed the heated oils for 16 weeks, starting on week 4. It is possible that the beneficial effect was conferred by the improvement in vascular response due to preserved plasma nitric oxide level. The groups that were fed the extract-treated oils also showed better organization of vascular elastic lamellae, smaller aortic tunica intima to tunica media ratio, and lower expression of vascular cell adhesion molecule-1 [63]. The findings indicated that the extract prevented oxidation in the oils upon heating, thus increasing their stability and antioxidant content. Heating was reported to reduce the antioxidant content of the oil, particularly reducing vitamin E levels [65]. Consumption of such oils prevented the development of hypertension due to preservation of vascular microstructure.

**Figure 5:**
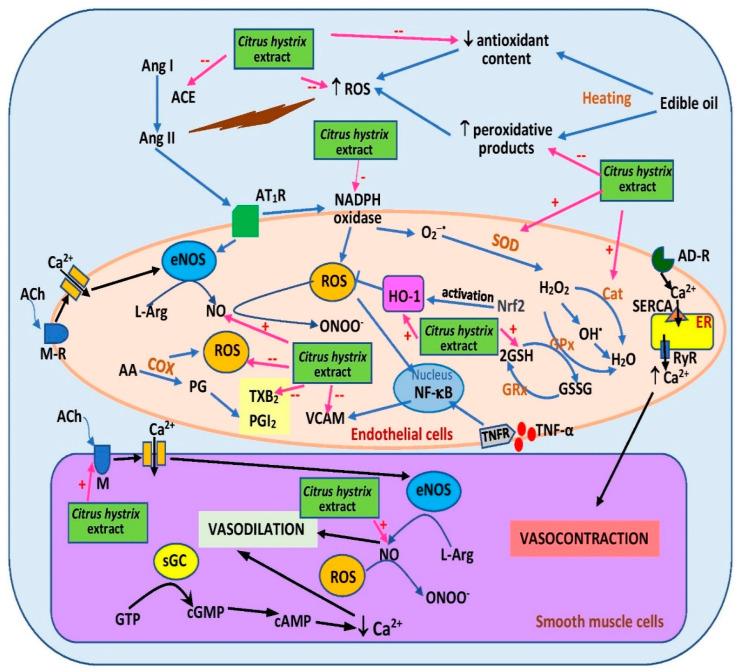
Possible sites of action of *C. hystrix* extract in ameliorating hypertension. AA, arachidonic acid; ACE, angiotensin-converting enzyme; ACh, acetylcholine; AD-R, adrenergic receptor; Ang I, angiotensin I, Ang II; angiotensin II; AT1R, angiotensin II type 1 receptor; cAMP, cyclic adenosine monoamine phosphate; cGMP, cyclic guanosine monophosphate; COX, cyclooxygenase; eNOS, endothelial nitric oxide synthase; ER, endoplasmic reticulum; GPx, glutathione peroxidase; FRx, glutathione reductase; GSSG, glutathione disulfide; GSH, reduced glutathione; GTP, guanosine-5′-triphosphate; HO-1, heme oxygenase-1; L-Arg, L-arginine; NF-κB, nuclear factor kappa-B; M-R, muscarinic receptor; NO, nitric oxide; PG, prostaglandin; PGI2, prostacyclin; ROS, reactive oxygen species; RyR, ryanodine receptor; SERCA, sarcoplasmic/endoplasmic reticulum Ca^2+^ ATPase; sGC, soluble guanylate cyclase; SOD, superoxide dismutase; TNF-α, tumor necrosis factor- α; TNFR, tumor necrosis factor receptor; TXB2, thromboxane B2; VCAM, vascular cell adhesion molecule; +, activates; --, inhibits.

Another study [64] investigated a similar treatment of frying oils with *C. hystrix* extract and enrichment with 2% cholesterol. The diet was then fed to ovariectomized female rats for six months. Consumption of the diet decreased blood pressure and was associated with reduced serum thiobarbituric acid reactive substance (TBARS) level (a lipid peroxidation indicator) and elevated serum antioxidant enzyme levels, namely superoxide dismutase and catalase, as well as serum glutathione level [64] (Figure 5).

The antihypertensive effects of the extract were further investigated via incorporation into rat diet [8,66]. Intake of a five-time-heated oil diet supplemented with 0.15% *C. hystrix* leaf extract for 16 weeks decreased systolic blood pressure in rats when compared with the unsupplemented group [8]. Reduced plasma TBARS, reduced serum ACE, and elevated serum heme oxygense-1 (HO-1) were also reported in the supplemented group [8]. HO-1 activation promotes vasodilation and diminishes oxidative stress [69]. Aortic rings obtained from the group also demonstrated reduced response to phenylephrine-induced vasoconstriction [66]. The beneficial effect on vasoconstriction was likely attributable to increased plasma NO level [66] and reduced plasma thromboxane B2 (a vasoconstrictor) [8] (Figure 5). The diet also preserved aortic histomorphometry, specifically intima–media area, intima–media thickness, and circumferential wall tension [8]. However, similar effects except for HO-1 were not observed in the group given ten-time-heated oil supplemented with the extract [8,66]. The extract did not affect plasma prostacyclin levels [8] and aortic response towards vasorelaxation triggered by acetylcholine and sodium nitroprusside [66]. The findings of the studies suggest that *C. hystrix* leaf extract supplementation promotes vasorelaxation by restoring the vasodilative properties and structure of blood vessels, likely via its antioxidant properties. The extract may also exert its effects via β- and α-adrenoceptor blockade, which should be further studied. Unfortunately, no clinical study was performed to confirm the effects seen in the animals. The effect of the extract on calcium handling proteins like sarcoplasmic/endoplasmic reticulum Ca^2+^ ATPase (SERCA), Na^+^/Ca^2+^ exchanger, L-type calcium channels, and ryanodine receptor 2 (RyR2) should be explored because intracellular calcium regulation is an important mechanism in vasoconstriction [70]. Other signaling pathways including the asymmetric dimethylarginine-NO (ADMA/NO) and RhoA/Rho kinase pathway, which mediates calcium sensitization, should be studied as well. ADMA is an inhibitor of NO synthesis [71].

Hypertension can cause end-organ damage in the kidneys and heart [72]. Dietary *C. hystrix* leaf extract (0.15%) reduced renal oxidative stress through reduction of TBARS content and NADPH oxidase activity [68]. NADPH oxidase is a superoxide anion generator [73]. Renal HO-1 was also increased in the rats that were fed a diet supplemented with the extract [68], while rats that were fed a diet containing 15% extract-treated heated oil plus 2% cholesterol had lower levels of cardiac C-reactive protein, TNF-α, troponin, and lactate dehydrogenase [67]. These findings suggest that *C. hystrix* leaf extract conserves the integrity of the kidneys and heart in rats fed a heated oil diet, likely by decreasing oxidative stress and inflammation in the organs. Further investigation of NADPH oxidase-4/H_2_O_2_/mTOR complex 1 (NOX4/H_2_O_2_/mTORC1) and PI3K/Akt signaling pathways should be conducted to validate the protective effects of the extract on hypertension-induced renal injury. The former pathway was reported to be activated, while the latter was suppressed in such models [74,75].

## 7. Effects on Cholesterol Level and Obesity

Hyperlipidemia is a metabolic disorder that can lead to the development of other metabolic disorders like hypertension and obesity. It arises from an imbalance in blood cholesterol levels: high in total cholesterol (TC) and low-density lipoprotein (LDL), and low in high-density lipoprotein (HDL) levels [76]. Many factors contribute to its pathogenesis including a high-fat diet and lack of physical activity [75]. Studies on *C. hystrix* have been conducted to assess its effects on the blood lipid profile (Table 5). A study by Nugraheni et al. [9] reported that plant rind or peel extract at 35, 70, and 140 mg/kg produced no significant change in serum LDL level in rats fed a high-fat diet. Conversely, another study by the same group [77] demonstrated a reduction in serum TC following administration of the extract; however, there was no significant increase in the cholesterol levels of the high-fat group as compared with the control group, suggesting that the hyperlipidemia model was not properly established in this study. Moreover, the investigators used the Least Significant Difference test as the post hoc test, which tends to give a false-positive result [78]. Therefore, the findings of the study are questionable and were not confirmative.

A study reported positive findings regarding the blood lipid profile of rats fed a high-fat diet following treatment with orally administered *C. hystrix* peel extract at 500 mg/kg [79]. The extract reduced serum TC, LDL, triglyceride (TG), and HDL in rats after 14 days of treatment. However, the baseline level for each blood parameter was not similar among the groups, which could have resulted in a false significant difference between groups. Sukalingam et al. [67] demonstrated that ovariectomized rats fed a high-fat diet containing 2% cholesterol and 15% heated oil supplemented with 1% *C. hystrix* extract during heating had reduced cardiac TG and free fatty acid levels after 6 months.

*C. hystrix* leaf aqueous extract in combination with galangal rhizomes and lemongrass extract produced similar effects to those of simvastatin (an antihyperlipidemic drug) on serum lipid profile and hepatic steatosis in rats fed a hypercholesterolemic diet [81]. However, it is difficult to ascertain the protective effect of the extract combination on hyperlipidemia because the comparison was made only against a positive control (simvastatin); there was no untreated hypercholesterolemic group to compare with. Moreover, the effectiveness of simvastatin in lowering blood cholesterol level was also not confirmed.

Obesity occurs due to imbalance in energy intake and expenditure. One strategy to reduce obesity is by increasing physical activity and reducing intake of calories. However, this approach is quite difficult to achieve in long-term. Therefore, natural products that can reduce the digestion and absorption of carbohydrates are sought out [82]. Only one study investigated the potential effect of *C. hystrix* on obesity. Watanabe et al. [80] reported that *C. hystrix* leaf ethanol extract at 100 μg/mL exhibited significant pancreatic lipase inhibition (>50%). Pancreatic lipase is an enzyme that converts TG to fatty acids and glycerol [82]. Two major compounds were then isolated from the extract: β-sitosterol and 3-[O-α-galactopyranosyl-(1”→6’)-O-β-galactopyranosyl]-1-O-linolenyl-2-O-palmityl-glyceride. Both compounds at 100 μg/mL demonstrated better inhibitory activity than the crude extract, resulting in 79% and 88% inhibition, respectively [80]. Based on these findings, the compounds should be researched further and developed as promising candidates for the management of obesity. The beneficial effects of the extract should be investigated in humans to affirm the findings in experimental animals.

**Figure 6:**
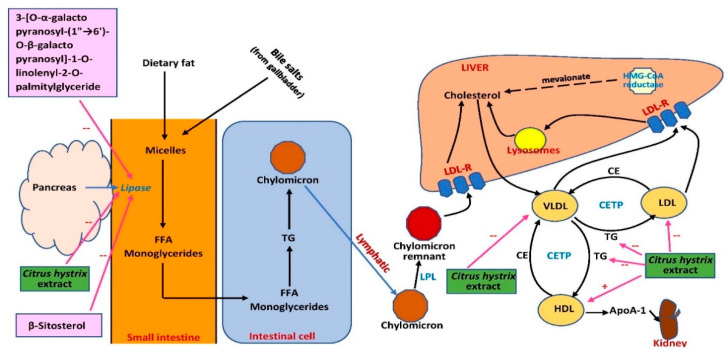
Possible sites of action of *C. hystrix* extract and its bioactive compounds in lipid metabolism. ApoA-1, apolipoprotein A-1; CE, cholesteryl ester; CETP, cholesteryl ester transfer protein; FFA, free fatty acid; HDL, high-density lipoprotein; HMG-CoA, 3-hydroxy-3-methylglutaryl-coenzyme A; LDL, low-density lipoprotein; LDL-R, low-density lipoprotein receptor; LPL, lipoprotein lipase; TG, triglyceride; VLDL, very-low-density lipoprotein; →, conversion or movement; --, inhibits.

Collectively, the effects of *C. hystrix* on serum blood lipids were not conclusive due to the unestablished models used in the studies. However, possible sites of action of *C. hystrix* extracts are summarized in Figure 6. More studies should be conducted using well-established models and appropriate experimental design, and possible mechanisms of blood lipid profile improvement should be elucidated. The effect of the extract on proteins involved in lipid metabolism and transport such as 3-hydroxy-3-methyl-glutaryl-coenzyme A reductase (HMG-CoA reductase), lecithin cholesterol acyltransferase, lipoprotein lipase cholesteryl ester transfer protein, and LDL receptor regulation should also be investigated. The effect of the extract on fatty acid oxidation mediated by peroxisome proliferator-activated receptors (PPARs) or possible inhibition of intestinal absorption of cholesterol should also be explored. In terms of its potential as an anti-obesity drug, the effects of the extract on lipid homeostasis should be investigated further. Lipogenesis-related proteins and genes including acetyl-CoA carboxylase 1 (ACC1), sterol regulatory element binding protein-1c (SREBP-1c), and fatty acid synthase (FASN) can be measured in white fat and liver. The effect of the extract on lipolysis can also be examined by analyzing the expression of hormone-sensitive lipase (HSL), adipose triglyceride lipase (ATGL), lipoprotein lipase (LPL), and β-oxidation-associated genes and proteins in the involved organs. Therefore, the extract and its phytochemical compounds have the potential to be developed as targeted therapies for obesity.

## 8. Pharmacokinetics and Safety

No published reports have been found on pharmacokinetics of *C. hystrix* extract or its bioactive compounds. Pharmacokinetic studies should be conducted to fully characterize the extract so that further studies can be appropriately designed.

Only two studies have been published regarding toxicity [83,84]. Orally administered *C. hystrix* chloroform and ethanol peel extract at 100–2500 mg/kg twice daily at various gestational days (days 2–5, 8–12, or 15 until labor) in rats resulted in dose-dependent interruption of implantation and abortion. Reduced fetal weight was observed in the group administered 1000 mg/kg of alcohol extract. However, these doses did not affect the rats’ estrus cycles [83]. The findings suggest that consumption of peel extract should be avoided in pregnancy. Nonetheless, further studies should be carried out to confirm these results.

The second study was a case report of a hiker who had applied the juice of *C. hystrix* onto his skin following advice from a friend to relieve the sting of insect bites and as an insect repellent [84]. He developed erythema and blisters at the sites of application. Based on this report, it can be presumed that *C. hystrix* has the potential to cause severe photodermatitis; although, it is difficult to validate this allergic reaction as no dosage or duration were documented. The lack of a significant sample size also warrants further investigation.

## 9. Conclusions and Directions for Future Study

*Citrus hystrix* contains bioactive compounds that have the potential to be developed as drug candidates for metabolic disorders. However, most of the studies conducted thus far were still at the initial stages of using crude extracts in vitro. In vivo studies should be conducted to confirm the observed in vitro effects. Bioactive compounds should be identified and isolated to pursue investigations of potential pharmacological activity. Regulation of the RAAS, which plays an important role in blood pressure control, by the extract or its bioactive compounds has not been thoroughly investigated. Other effects on hypertension that can be studied are the kallikrein-kinin and NO-cyclic guanosine monophosphate (NO/cGMP) pathways. In addition, AMP-activated protein kinase/sirtuin 1 (AMPK/SIRT1), fibroblast growth factor-19/21 (FGF19/21), Janus kinase/signal transducer and activator of transcription (JAK/STAT), and Notch signaling pathways implicated in obesity could also be investigated. Activation of the stimulator of interferon genes/interferon regulatory factor 3 (STING/IRF3) pathway that causes inflammation and apoptosis in organs is implicated in pancreatic β-cell lipotoxicity; therefore, the potential protective effects of the plant against β-cell damage in this pathway can be investigated. To date, only animal studies have been conducted to assess the pharmacological properties of *C. hystrix* and its bioactive compounds. Lack of clinical studies could restrict further development of the compounds as drug candidates for metabolic disorders. To progress to clinical trials, the pharmacokinetics and toxicity of the crude extract and bioactive compounds must be elucidated.

## Figures and Tables

**Table 1:** Traditional medicinal uses of *C. hystrix*.

Plant Parts	Medicinal Uses	Reference
Rinds	Worm infestation Headache	[10]
Fruit juice	Cancer	[16,17]
	Skin diseases	
	Dandruff	
	Hair loss	
Fruit juice and rinds	An ingredient in *jamu* for promoting physical and general health	[10]
Fruits	Stomachache	[11]
Leaves and fruits	Steam bath Aphrodisiac Diabetes mellitus Fever Rheumatism	[13,14]
	Flu Hypertension Diarrhea Heart disease Dizziness Indigestion	
Leaves	Stomachache	[10,18]
	To maintain healthy teeth and gums Scurvy	
Roots and leaves	Hypertension	[15]

**Table 2:** Phytochemical compounds in various parts of *C. hystrix*.

Plant Part	Type	Phytochemical	Potential Activity *	Reference
Leaves	Monoterpenoids	Citronellal	Antimicrobial	[20]
		Linalool oxide		[21]
		Citronellol		
		Terpeneol		
		Geraniol		
		α-Thujene		
		α-Cubene		
		β-Cubene		
	Diterpene	Phytol	Antileukemic	[22]
	Triterpene	Lupeol	Antileukemic	[21,22,23]
		Agrostophillinol	Anti-inflammatory	[18]
		Squalene		[21]
	Tetranortriterpenoids (limonins)	Limonexin		[24]
		Limonexic acid		
		Limonin	Antibacterial	
	Sesquiterpenes	Spathulenol		[21]
		Nerolidol		
		Germacrene		
		Caryophyllene oxide		
		Longipinenepoxide		
		α-Cedrane		
		Globulol		
	Phytosterols	Stigmasterol		
		Sitosterol		
		Lanost-7-en-3-one		
	Glyceroglycolipids	l,2-di-O-a-linolenoyl-3-O-galactopyranosyl-sn-glycerol l-O-a-Linolenoyl-2-O-palmitoyl-3-O-galactopyranosyl-sn-glycerol	Viral tumor- promoting inhibitors	[25]
	Phenol	α-Tocopherol	Antioxidant	[21,26]
	Flavonoids	Cyanidin	Antioxidant	[27,28]
		Myricetin		
		Peonidin		
		Quercetin		
		Luteolin		
		Hesperetin		
		Apigenin		
		Isorhamnetin		
		Hesperidin		[7]
		Diosmin		
		Apiin		
		Saponarin		
		Apigetrin		
		Rutin		
		Eriodictyol rutinoside		[28]
		Eriodictyol neohesperidoside		
		Phloretin		
		Diosmetin		
Roots	Coumarins	Hystrixarin		[7,24,29]
		Hopeyhopin		
		Peroxytamarin		
		Tamarin	Antibacterial	
		Trans-decursidinol		
		Suberosin		
		Scoparone		
		Scopoletin		
		Umbelliferone		
		cis-Khellactone		
		Oxypeucedanin hydrate		
		cis-Casegravol		
		Citrusarin A-B		
		Seselin		
		5-Hydroxy-seselin		
		Dipetalolactone		
		Xanthyletin		
		Osthenol		
		Isoimperatorin		
		Xanthotoxol		
	Benzenoid	Hystroxene-I		
	Quinolinone	Hystrolinone 1-Methyl-2-n-nonyl-4-quinolinone		
	Cinnamyl alcohol	Etrogol		
	Alkyl phenylketones	Xanthoxylin (Brevifolin)	Antibacterial	
	Flavonoids	Crenulatin		
		Yukovanol	Antibacterial	
		P-hydroxy-benzaldehyde		
	Acridone	Baiyumine-A		
		5-Hydroxy-noracronycine	Anti-HIV-1 protease Antioxidant	
		Citracridone-I		
		Citpressine-I		
		Citramine		
	Benzoic acid	Valencic acid		
		p-Hydroxybenzoic acid		
	Lignan	Syringaresinol	Antioxidant Antibacterial	
	Suberin	20,30-dihydroxydihydrosuberin		
	Phenolic	Vanillic acid		
		Tyrosol		
Rinds	Coumarins	Citrusosides B–D	Anticholinesterase	[30]
		Oxypeucedanin methanolate		[31]
		Oxypeucedanin hydrate		
		Isoimperatorin		
		Oxypeucedanin		
		Pabulenol		
		Bergamottin		
		Hydroxy-7′-methoxy-bergamottin		
		6′,7′-dihydroxy-bergamottin		
		7-hydroxycoumarin		
		Umbelliferone	α-Amylase inhibitor	[32]
		Bergamottin		[33]
		Oxypeucedanin		
		Citrusoside E-G		
		Citrusoside H	Anticholinesterase	
		Citrusoside I-O		
		Bergaptol	Antioxidant Anticholinesterase Antitumor α-Amylase inhibitor	[32]
		Isoimperatorin	Antioxidant Antitumor α-Amylase inhibitor	
	Pectin	Anhydrouronic acid (85%)		[34]
	Monoterpenes	β-pinene Limonene Sabinene	Antimicrobial	[20]
	Tetranor-triterpenoids	Limonin	Metal chelator α-Amylase inhibitor	[32]
	Acridone	Citracridone I	Antioxidant Metal chelator Antitumor α-Amylase inhibitor	
	Phytosterol	Daucosterol	Antioxidant Antitumor α-Amylase inhibitor	
		Stigmasterol	Antioxidant Metal chelator Antitumor α-Amylase inhibitor	
	Flavonoids	Trihydroxypyranoflavone	α-Amylase inhibitor Anticholinesterase Antitumor	
		Dimethyl-6-prenylpyranoflavone	Anticholinesterase Antitumor α-Amylase inhibitor	
		Tangeretin	Antioxidant Antitumor α-Amylase inhibitor	
		Nobiletin	Antioxidant Metal chelator Antitumor α-Amylase inhibitor	
		Tetramethoxyflavone	Antioxidant Metal chelator Antitumor α-Amylase inhibitor	
		Natsudaidain	Antioxidant Metal chelator	
		Quercetin		[19]
		Hesperidin		
		Hesperitin		[33]
Fruits	Coumarins	Bergamottin	Nitric oxide synthesis inhibitor, antitumor	[25,31]
		Oxypeucedanin	Nitric oxide synthesis inhibitor	
		5-[(6′,7′-Dihydroxy-3′,7′-dimethyl-2-octenyl)oxy]psoralen		
Twig Essential oil	Monoterpenes	β-citronellal		[35]
		4-Terpeneol		
		L-linalool		
		β-Citronellol		
		Citronelyl acetate		
		Sabinene		
		β-Pinene		
		β-Micrene		
		Tran-β-ocimene		
		(-)-Isopulegol		
		Cis-Linalol oxide		
	Sesquiterpenes	Trans-β-caryopilene		
		Nerolidol		
Leaf essential oil	Monoterpenes	α-Pinene		[36,37,38,39]
		Camphene		
		β-Pinene		
		β-Myrcene		
		α-Phellandrene		
		β-Phellandrene		
		β-Car-3-ene		
		p-Cymene		
		Limonene		
		1,8-Cineole		
		p-Mentha-2,4(8)-diene		
		Linalool		
		endo-Fenchol		
		cis-p-Menth-2-en-ol		
		Sabinene		
		α-Terpinene		
		(E)-β-Ocimene		
		γ-Terpinene		
		Terpinolene		
		trans-p-Menth-2-en-ol		
		p-Menth-8-en-3-ol		
		Citronellal		
		Geranial		
		Isopulegol		
		Isoneopulegol		
		Borneol		
		Terpinen-4-ol		
		p-Cymen-8-ol		
		Geranyl acetate		
		α-Terpineol		
		Myrtenol		
		trans-Piperitol		
		Citronellol		
		Geraniol		
		Bornyl acetate		
		Carvacrol		
		Citronellyl acetate		
		Neryl acetate		
		trans-p-Menth-6-ene-2,8-diol		
	Sesquiterpenes	α-Copaene		
		cis-Cadina-1,4-diene		
		Hedycaryol		
		Caryophyllene oxide		
		Cubenol		
		β-Eudesmol		
		(E)-β-Caryophyllene		
		trans-α-Bergamotene		
		α-Humulene		
		Germacrene D		
		Bicyclogermacrene		
		(E,E)-α-Farnesene		
		β-Bisabolene		
		δ-Cadinene		
		(E)-Nerolidol		
		Spathulenol		
		Globulol		
Rind essential oil	Monoterpenes	α-Thujene		[38]
		α-Pinene		
		Camphene		
		Sabinene		
		β-Pinene		
		Myrcene		
		p-Cymene		
		β-Phellandrene		
		Limonene		
		*trans*-Linalool oxide		
		Linalool		
		Citronellal		
		Terpinen-4-ol		
		α-Terpineol		
		*trans*-Carveol		
		Citronellol		
		Geranyl acetate		
	Sesquiterpenes	α-Copaene		
		β-Elemene		
		δ-Cadinene		
	Ester	Hexyl hexanoate		

* The activity that was screened by the respective studies.

**Table 3:** Studies of *C. hystrix* on diabetes mellitus.

Plant Extract	Model	Outcomes	Study
Rinds and pulps (Powdered form)	In vitro	↑ glucose adsorption capacity ↓ glucose dialysis retardation index ↓ starch digestibility	[43]
Rinds (Ethyl acetate fraction and water residue)	In vitro	↓ α-amylase activity ↓ α-glucosidase activity ↓ Starch digestibility	[44]
Fresh fruit juice	In vitro	↓ α-amylase activity ↓ α-glucosidase activity Almost comparable to acarbose	[45]
Rinds (Ethanol)	In vitro	↓ α-amylase activity	[46]
Leaves (Ethanol)	In vivo STZ-induced diabetes in rats	Extract at 150 and 300 mg/kg bw in drinking water for 8 weeks: ↓ fasting blood glucose ↓ cataract incidence ↓ oxidative stress and inflammation ↓ vascular leakage	[7]
Fruit juice in combination with other extracts (functional drink)	In vivo STZ-induced diabetes in rats	Functional drink (18.2 mL/kg bw) for 14 days: ↓ fasting blood glucose ↑ pancreatic β-cell viability ↑ Langerhans islet viability	[47]

bw, body weight; STZ, streptozotocin, ↓, reduced; ↑, increased.

**Table 4:** Effects of *C. hystrix* extract on hypertension and related end-organ damage.

Plant Extract	Model	Mode of Administration	Outcomes	Study
Leaves (Aqueous)	In vitro	-	Good ACE-inhibiting activity (~90%)	[59]
Leaves (Ethanol)	OVX rats fed a high-fat diet for 6 months	Addition of extract into the frying oil (1%)	Both in 5HPO and 10HPO groups: ↓ PV in oil, ↓ BP, ↓ serum oxidative product, ↑ serum antioxidant enzymes	[64]
Leaves (Ethanol)	Heated oil diet-induced in rats for 16 weeks	Addition of extract into the frying oil (1%)	Both in 5HPO and 10HPO groups: ↓ PV and ↑ TPC in oil ↓ BP, ↑ plasma NO, ↓ vasoconstriction response to PE ↑ vasorelaxation response to ACh and SNP, ↓ TI/TM. ↓ VCAM	[63]
Leaves (Ethanol)	Heated oil diet-induced in rats for 16 weeks	Dietary (0.15%)	In 5HPO group but not in 10HPO group: ↓ BP, ↓ plasma TBARS, ↓ serum ACE, ↓ plasma TXB_2_, ↓ IMA, ↓ IMT, ↓ CWT In both 5HPO and 10HPO groups: ↑ serum HO-1, ↔ plasma PGI_2_	[8]
Leaves (Ethanol)	Heated oil diet-induced in rats for 16 weeks	Dietary (0.15%)	In 5HPO group but not in 10HPO group: ↑ plasma NO, ↓ vasoconstriction response to PE ↔ vasorelaxation response to ACh and SNP	[66]
Leaves (Ethanol)	OVX rats fed a high-fat diet for 6 months	Addition of extract into the frying oil (1%)	In both 5HPO and 10HPO groups: ↓ serum CRP, ↓ serum TNF-α, ↓ cardiac troponin, ↓ cardiac LDH	[67]
Leaves (Ethanol)	Heated oil diet-induced in rats for 16 weeks	Dietary (0.15%)	Both 5HPO and 10HPO groups: ↓ renal TBARS, ↓ renal NOX, ↑ renal HO, ↓ serum creatinine in 5HPO group	[68]

ACE, Angiotensin-converting enzyme; ACh, acetylcholine; BP, blood pressure; CRP, C-reactive protein; CWT, circumferential wall tension; HO-1, heme oxygenase-1; IMA, intima-media area; IMT, intima-media thickness; LDH, lactate dehydrogenase; NO, nitric oxide; NOX, NADPH oxidase; OVX, ovariectomized; PE, phenylephrine; PGI2, prostacyclin; PV, peroxide value SNP, sodium nitroprusside; TBARS, thiobarbituric acid reactive substance; TI/TM, ratio of tunica intima to tunica media; TNF-α, tumor necrosis factor-α; TPC, total phenolic content; TXB2, thromboxane B2, VCAM-1, vascular cell adhesion molecule-1; 5HPO, five-time-heated palm oil; 10HPO, ten-time-heated palm oil. ↑, increased; ↓, decreased; ↔, no change.

**Table 5:** Effects of *C. hystrix* and its phytochemical compounds on hyperlipidemia and organ cholesterol level.

Plant Extract	Model	Mode of Administration	Outcomes	Study
Rinds (Ethanol)	A high-fat diet- induced hyperlipidemia in rats	Oral (35, 70, and 140 mg/kg bw) for 3 weeks	No significant change was noted in serum LDL level	[9]
Rinds (Ethanol)	A high-fat diet- induced hyperlipidemia in rats	Oral (35, 70, and 140 mg/kg bw) for 3 weeks	Extract at 70 and 140 mg/kg: ↓ serum TC	[77]
Rinds (Methanol)	A high-fat diet- induced hyperlipidemia in rats	Oral (500 mg/kg) for 14 days	↓ serum TC ↓ serum TG ↑ serum HDL ↓ serum LDL	[79]
Leaves (Ethanol)	OVX rats fed a high-fat diet for 6 months	Addition of extract into the frying oil (1%)	↓ cardiac free fatty acid ↓ cardiac TG	[67]
Leaves (Ethanol)	In vitro	-	Pancreatic lipase activity inhibition: Extract: 58%. Isolate β-sitosterol (100 μg/mL): 79% Isolate 3-[O-α-galactopyranosyl-(1”→ 6’)-O-β- galactopyranosyl]-1-O-linolenyl-2-O-palmitylglyceride (100 μg/mL): 88%	[80]
Leaves in combination with galangal rhizomes and lemongrass extracts (Aqueous)	Hyper- cholesterolemic diet (3% cholesterol) in rats	Oral mixed extract at 400 mg/kg/day for 28 days	Compared to simvastatin-treated group: ↔ serum lipid profile ↔ centrilobular steatotis ↔ periportal steatosis ↔ hepatitis	[81]

HDL, high-density lipoprotein; LDL, low-density lipoprotein; OVX, ovariectomized; TC, total cholesterol; TG, triglyceride, ↑, increased; ↓, decreased; ↔, no change.

## Data Availability

No new data were created or analyzed in this study. Data sharing is not applicable to this article.
